# Low-dose ATG/PTCy for graft-versus-host disease prevention in haploidentical transplantation: a single-center experience

**DOI:** 10.3389/fonc.2025.1569149

**Published:** 2025-06-09

**Authors:** Jian Hong, Xinglin Liang, Jing Ni, Min Ruan, Zhangbiao Long, Jifei Dai, Li Liang, Mingya Yang, Ziyang Zhang, Shihao Zhang, Jian Ge, Mingzhen Yang, Qingsheng Li

**Affiliations:** ^1^ Department of Hematology, the First Affiliated Hospital of Anhui Medical University, Hefei, China; ^2^ Department of Hematology, Xuanwu Hospital, Capital Medical University, Beijing, China; ^3^ Institute of Clinical Pharmacology, Anhui Medical University, Key Laboratory of Anti-Inflammatory and Immune Medicine, Ministry of Education, Anhui Collaborative Innovation Centre of Anti-Inflammatory and Immune Medicine, Hefei, China

**Keywords:** anti-thymocyte globulin, post-transplant cyclophosphamide, graft-versus-host disease, haploidentical hematopoietic stem cell transplantation, absolute lymphocyte count

## Abstract

**Background:**

The combination of anti-thymocyte globulin (ATG) and post-transplant cyclophosphamide (PTCy) has been administered for graft-versus-host disease (GVHD) prophylaxis of haploidentical hematopoietic stem cell transplantation (haplo-HSCT) in recent years. Varied doses of ATG and PTCy were applied in multiple studies with promising outcomes.

**Methods:**

We retrospectively analyzed 51 consecutive leukemia patients who underwent haplo-HSCT with the joint use of low-dose ATG (27 patients with 7.5 mg/Kg and 24 patients with 5 mg/Kg) and PTCy (29 mg/Kg) for GVHD prophylaxis in our center. The impact of different ATG doses and absolute lymphocyte count (ALC) before ATG infusion was also evaluated.

**Results:**

The 100-day cumulative incidences (CIs) of grade I-IV, II-IV and III-IV acute GVHD of the whole cohort were 42.9%, 34.7% and 12.2%, respectively. The 2-year CIs of overall and moderate-to-severe chronic GVHD were 44.7% and 27.7%, respectively. The 2-year overall survival, disease-free survival, non-relapse mortality and CI of relapse were 66.7%, 54.8%, 25.5% and 19.7%, respectively. Between 7.5 and 5 mg/Kg ATG groups, no significant difference on CIs of acute GVHD was observed. Interestingly, pre-ATG ALC impacted the occurrence of acute GVHD. With a cutoff point of 0.585×109/L, low ALC group showed reduced CIs of grade I-IV (16.7% versus 58.0%, p=0.01), II-IV (16.7% versus 45.1%, p=0.06) and III-IV (0 versus 19.4%, p=0.05) acute GVHD as compared to high ALC group.

**Conclusions:**

The results suggested that this low-dose ATG/PTCy regimen was feasible and pre-ATG ALC levels could influence the occurrence of acute GVHD in this regimen.

## 1 Introduction

During the past 20 years, the outcome of haploidentical hematopoietic stem cell transplantation (haplo-HSCT) has been dramatically improved, thanks to effective graft-versus-houst disease (GVHD) prophylaxis strategies with anti-thymocyte globulin (ATG) and post-transplant cyclophosphamide (PTCy). In China, ATG-based regimen is much more widely used. Compared to identical sibling HSCT, haplo-HSCT with ATG may achieve similar overall survival (OS) and disease-free survival (DFS) in acute myeloid leukemia (AML) patients, but incidences of acute and chronic GVHD were still significantly higher (1). In addition, the application of ATG is usually associated with higher risk of infections, especially virus reactivation (2–5).

In recent years, the combination of ATG and PTCy was reported to effectively reduce GVHD in HSCT patients. Low-dose PTCy (14.5 mg/Kg for 2 days) was demonstrated to augment the protective effect of ATG on GVHD and boost the reconstitution of regulatory T cells in a HSCT mouse model (6). In subsequent prospective trials for haplo-HSCT with maternal or collateral relative donors, the group with low-dose PTCy plus standard-dose ATG (2.5 mg/Kg for 4 days) showed significantly lower incidences of GVHD and comparable relapse and OS, as compared to ATG alone (6, 7). Besides, the joint use of ATG and PTCy at other doses and timings was also implemented in multiple studies with haplo- or unrelated donor-HSCT, resulting in promising outcomes (8–14).

Here, we report the low-dose ATG/PTCy regimen for haplo-HSCT in our center, which includes 2.5 mg/Kg ATG for 2 or 3 days (from day -4 or -3 to day -2) and 14.5 mg/Kg PTCy for 2 days (day +3 and +4). In addition, the impact of different total ATG doses (7.5 mg/Kg versus 5 mg/kg) and absolute lymphocyte count (ALC) before ATG infusion was analyzed in attempt to further optimize this ATG/PTCy regimen.

## 2 Methods

### 2.1 Patient selection

We included 51 consecutive leukemia patients who underwent haplo-HSCT with the low-dose ATG/PTCy regimen in the First Affiliated Hospital of Anhui Medical University, Hefei, China, from February 2018 to March 2022. All patients had a Karnofsky Performance Score (KPS) >60%. Clinical features, post-transplant outcomes and adverse events were collected retrospectively. This study was conducted in accordance with the Declaration of Helsinki and approved by the Hospital Ethics Committee. Informed consent was obtained from patients or their legal guardians before HSCT.

### 2.2 HLA typing and donors

High-resolution molecular typing for human leukocyte antigen (HLA)-A, -B, -C, -DRB1, -DQB1 and –DPB1 was done for both recipients and donors. Young and male donor is the first choice for the transplant. Donors were mobilized with granulocyte colony-stimulating factor (7.5-10 μg/kg per day), and collection of bone marrow/peripheral blood stem cells or both, which usually took one or two days, started from the 5th day of mobilization. A minimum dose of 2×10^6^ CD34^+^ cells/Kg recipient body weight was required for the transplant.

### 2.3 Transplant procedures

The conditioning regimen consisted of the following: busulfan (BU), 3.2 mg/kg intravenously for 4 days from day -7 to -4; cyclophosphamide (Cy), 50 mg/Kg intravenously for 2 days from day -3 to -2; rabbit ATG (thymoglobulin; Genzyme-Sanofi, Lyon, France), 2.5 mg/Kg for 2 or 3 days from day -4 or -3 to day -2; simustine, 250 mg/m^2^ orally on day -8 for all acute lymphoid leukemia (ALL) patients and a proportion of AML patients. Two doses of 14.5 mg/Kg Cy were given on days +3 and +4. In addition, patients received cyclosporine, mycophenolate mofetil and short-term methotrexate for GVHD prophylaxis. Cyclosporine was administered at a dose of 2.5 mg/Kg intravenously from day -2 and adjusted to achieve a therapeutic level of 200 to 300 μg/L. Cyclosporine taping started around day +60 to +75 for all patients without GVHD. Mycophenolate mofetil was given orally at a dose of 0.5g q12h from day -2 and discontinued on around day +30 if no acute GVHD was present.

Granulocyte colony-stimulating factor (5 μg/kg) and recombinant human thrombopoietin (300 U/Kg) were administered subcutaneously from day +5. Prophylactic anti-bacterial (levofloxacin), anti-fungal (micafungin or voriconazole), anti-viral (ganciclovir from day -7 to -2, and acyclovir after stem cell infusion) and anti-pneumocystis Jiroveci pneumonia (oral septra) therapies were administered to all patients. Pre-emptive therapy with ganciclovir was given once cytomegalovirus (CMV) deoxyribonucleic acid (DNA) was > 1000 IU/ml by quantitative polymerase chain reaction (PCR). Rituximab was pre-emptively given once Epstein–Barr virus (EBV) DNA was > 10^5^ IU/ml.

### 2.4 Engraftment and chimerism analysis

Neutrophil engraftment was defined as the first of 3 consecutive days when absolute neutrophil count exceeds 0.5×10^9^/L, and platelet engraftment was defined as the first day of 7 consecutive days with a platelet count more than 20×10^9^/L without platelet transfusion. The whole blood and leukocyte chimerism after the transplant was analyzed using PCR of short tandem repeats.

### 2.5 Statistical analysis

The last follow-up was updated in March 2024. Patient characteristics were reported as descriptive statistics. Chi-square and Fisher’s exact tests were used to compare categorical variables between two ATG groups, while the Mann-Whitney test was used for continuous variables.

Competing risk analysis was applied for the calculation of cumulative incidences (CIs) of GVHD, relapse (CIR), non-relapse mortality (NRM) and CMV/EBV viremia. Death from any cause was treated as the competing risk for acute GVHD, chronic GVHD and CMV/EBV viremia, while relapse and NRM were as the competing risk for each other. Excluding two patients with graft failure, 49 patients were eligible for acute GVHD evaluation. Forty-seven patients survived for more than 100 days after transplant and were eligible for chronic GVHD evaluation. Kaplan-Meier method was applied to estimate DFS and OS. Receiver operating characteristic (ROC) analysis was performed to determine an optimal cutoff value of ALC for acute GVHD of any grade.

All p-values are two-tailed, and *P* < 0.05 was considered statistically significant. Statistical analyses were performed using SPSS version 20.0 (SPSS, Inc. Chicago, IL, NY) and R software version 3.5.3 (http://www.r-project.org).

## 3 Results

### 3.1 Patient characteristics

Characteristics of total 51 patients are summarized in **Table 1**. The median age of all patients at transplantation was 39 years (range, 14–58 years). Thirty-one patients were diagnosed with acute myeloid leukemia, 17 with acute lymphoblastic leukemia/lymphoblastic lymphoma and 2 with chronic myelogenous leukemia. The median follow-up was 858 days (range, 25–2209 days). Before December of 2020, we applied 7.5 mg/Kg of ATG in our transplant protocol, and thereafter changed the dose of ATG to 5 mg/Kg. In total, 27 patients received 7.5 mg/Kg ATG and 24 patients received 5.0 mg/Kg ATG for GVHD prophylaxis. The median follow-up was 1359 days (range, 25–2209 days) and 792 days (range, 55–1157 days) for 7.5 mg/Kg and 5 mg/Kg ATG groups, respectively.

**Table 1:** Patient characteristics.

Characteristics	All patients (N=51)
Median age at HSCT (range), year	39 (14-58)
Gender	
Male	31
Female	20
Disease type	
Acute myeloid leukemia	31
Acute lymphoblastic leukemia/lymphoblastic lymphoma	17
Chronic myelogenous leukemia	3
Disease status at transplantation	
CR1/CP1	35
CR2/CP2	13
Non-remission	3
Disease risk index	
Low/intermediate	40
High/very high risk	8
Not available	3
Median time from diagnosis to transplantation (range), month	9.0 (5.0-92.0)
Donor-patient sex matched	
Male to male	22
Male to female	17
Female to male	9
Female to female	3
ABO matched	
Matched	28
Major mismatched	9
Minor mismatched	14
Donor-patient relation	
Father donor	12
Mother donor	2
Sibling donor	11
Children donor	26
ATG total dose	
7.5mg/Kg	27
5mg/Kg	24
HLA match	
6/12	32
7/12	17
8/12	0
9/12	2
Graft type	
BM+PB	26
BM	2
PB	23
Median nucleated cells, ×10^8^ cells per kg (range)	7.63 (2.79-18.47)
Median CD34^+^ cells, ×10^6^ cells per kg (range)	3.63 (1.45-15.10)
Median follow-up, days (range)	858 (25-2209)

ATG, anti-thymocyte globulin; BM, bone marrow; CR, complete remission; CP, chronic phase; HLA, human leukocyte antigen; HSCT, hematopoietic stem cell transplantation; PB, peripheral blood.

### 3.2 Hematopoietic recovery, regimen-related toxicity and virus reactivation

Median time to neutrophil and platelet engraftment was 11 days (range, 9–26 days) and 12 days (range, 9–135 days), respectively. In total, 1 patient had primary graft failure, 1 had secondary graft failure and 2 patients had primary delayed platelet engraftment. The patient with primary graft failure received 7.5 mg/Kg ATG and had positive class I donor-specific anti-HLA antibody (mean fluorescence intensity 8447) and desensitization treatments were performed before the transplant. The patient with secondary graft failure, who received 7.5 mg/Kg ATG, was possibly precipitated by virus infections (CMV and human herpesvirus 6).

Hemorrhagic cystitis occurred in 20 patients with 3 of grade III-IV. Transplant-associated thrombotic microangiopathy and capillary leak syndrome were documented in 1 and 2 patients, respectively. By day 180 after transplant, 4 patients had fungal infection. The 180-day CIs of CMV and EBV reactivation were 42.0% (95%CI, 28.1%-55.3%) and 62.7% (95%CI, 47.7%-74.6%), respectively. CMV disease was observed in 2 patients, presenting as CMV pneumonia in one case and CMV enteritis in the other. No post-transplant lymphoproliferative disorder was developed.

### 3.3 GVHD and transplant outcomes

**Figure 1:**
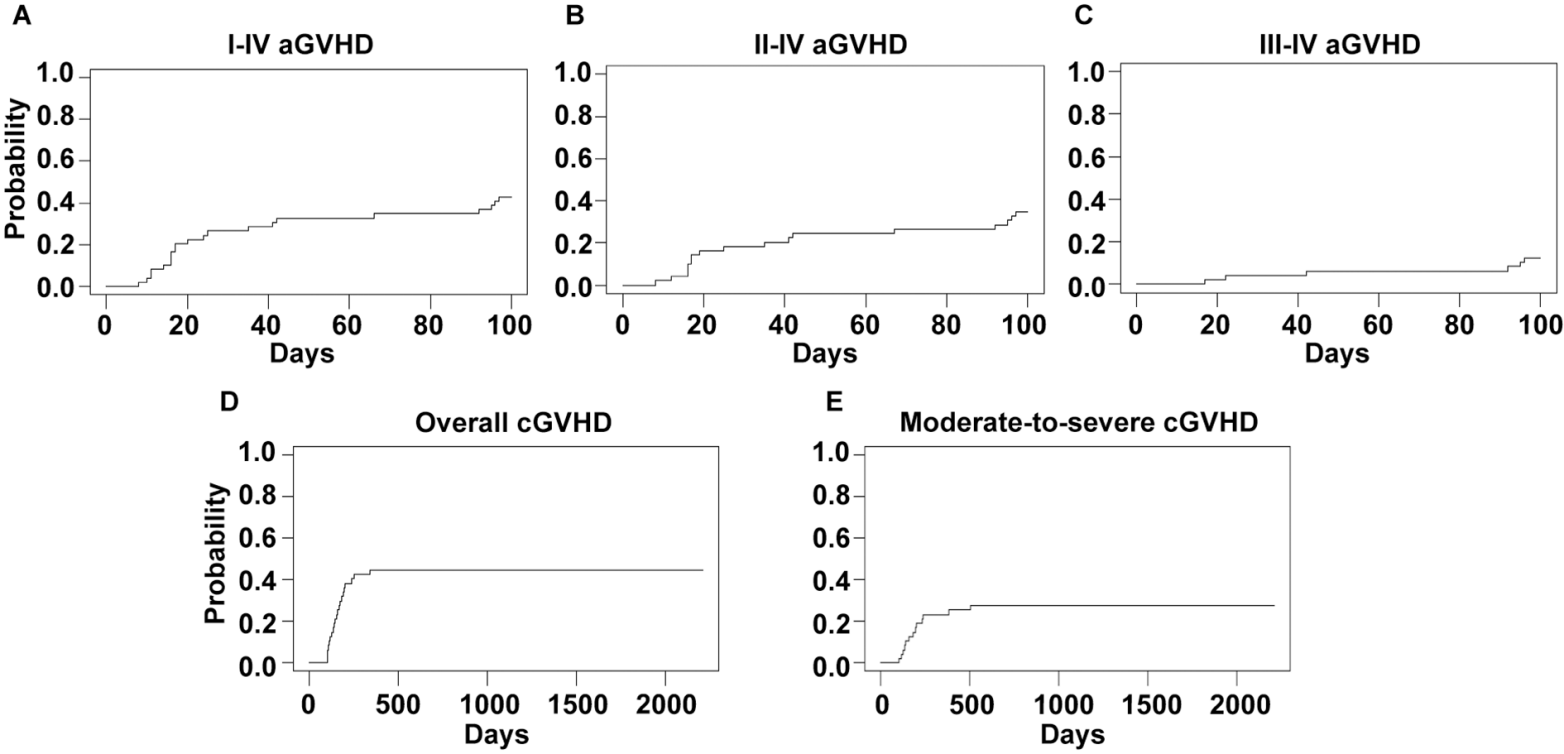
GVHD incidences of the whole cohort. Cumulative incidences of I-IV **(A)**, II-IV **(B)** and III-IV **(C)** acute GVHD and overall **(D)** and moderate-to-severe **(E)** chronic GVHD are shown.

The 100-day CIs of grade I-IV, II-IV and III-IV acute GVHD were 42.9% (95%CI, 28.8%-56.9%), 34.7% (95%CI, 21.2%-48.2%) and 12.2% (95%CI, 3.0%-21.5%), respectively (**Figures 1A–C**). The median time to onset of grade I-IV acute GVHD was 22 days (range, 8–97 days). Forty-seven patients survived for more than 100 days after transplant and were eligible for chronic GVHD evaluation. The 2-year CIs of overall and moderate-to-severe chronic GVHD were 44.7% (95%CI, 30.2%-59.1%) and 27.7% (95%CI, 14.7%-40.6%), respectively (**Figures 1D, E**). The median time to onset of chronic GVHD was 154 days (range, 100–341 days).

**Figure 2:**
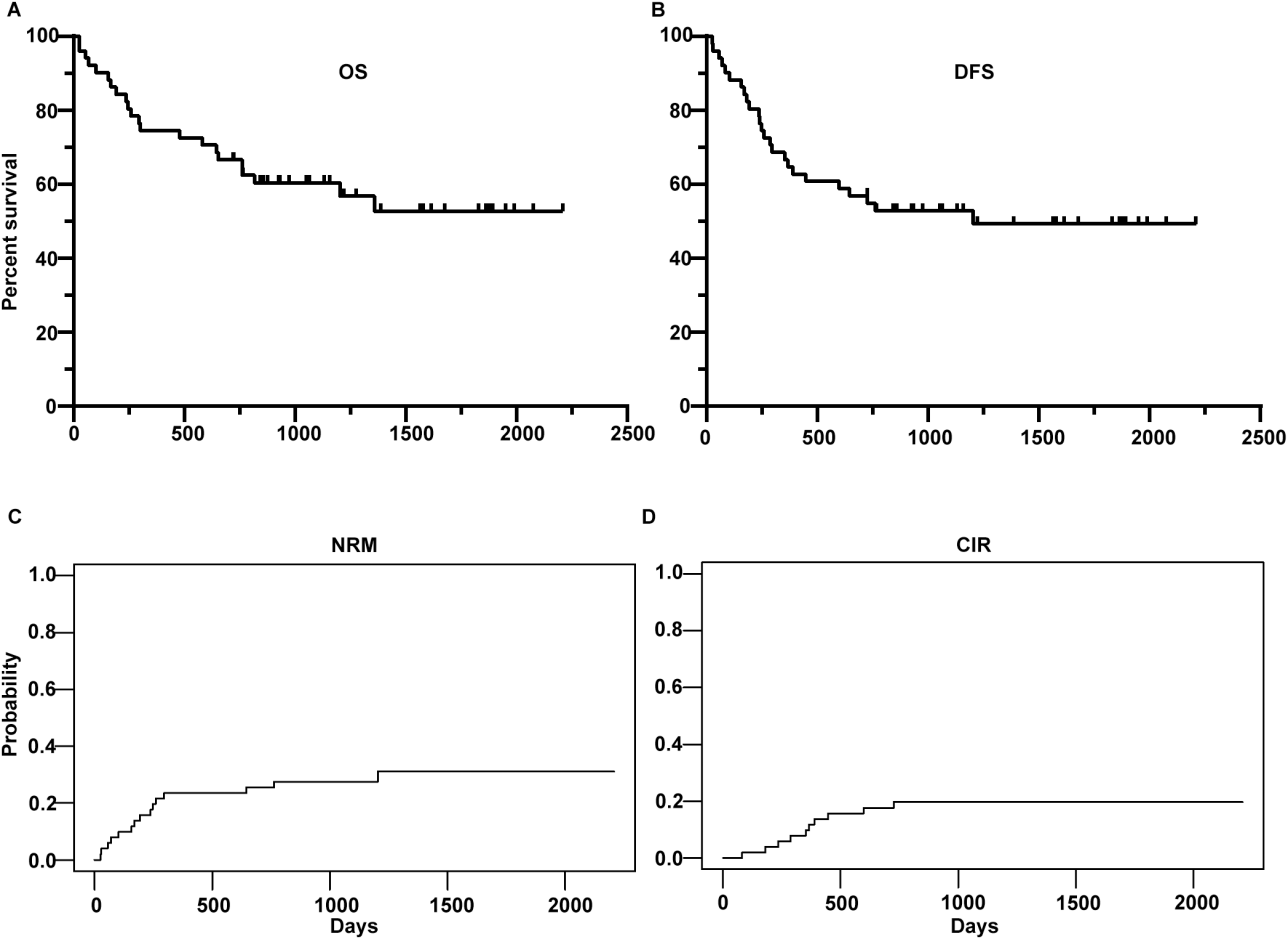
Overall survival (OS) **(A)**, disease-free survival (DFS) **(B)**, non-relapse mortality (NRM) **(C)** and cumulative incidence of relapse (CIR) **(D)** of the whole cohort.

The 2-year OS, DFS, NRM and CIR were 66.7% (95%CI, 54.9- 80.9%), 54.8% (95%CI, 42.7-70.4%), 25.5% (95%CI, 13.4%-37.6%) and 19.7% (95%CI, 8.6%-30.8%), respectively (**Figure 2**). Up to the final follow-up, 10 patients relapsed. The median time to relapse was 359 days (range, 82–726 days). Fourteen patients experienced NRM. Causes of death were relapse in 8 (36%) patients, GVHD in 7 (32%) patients, infection in 5 (23%) patients and graft failure in 2 (9%) patients.

### 3.4 Effect of ATG doses and pre-ATG absolute lymphocyte count on GVHD

**Figure 3:**
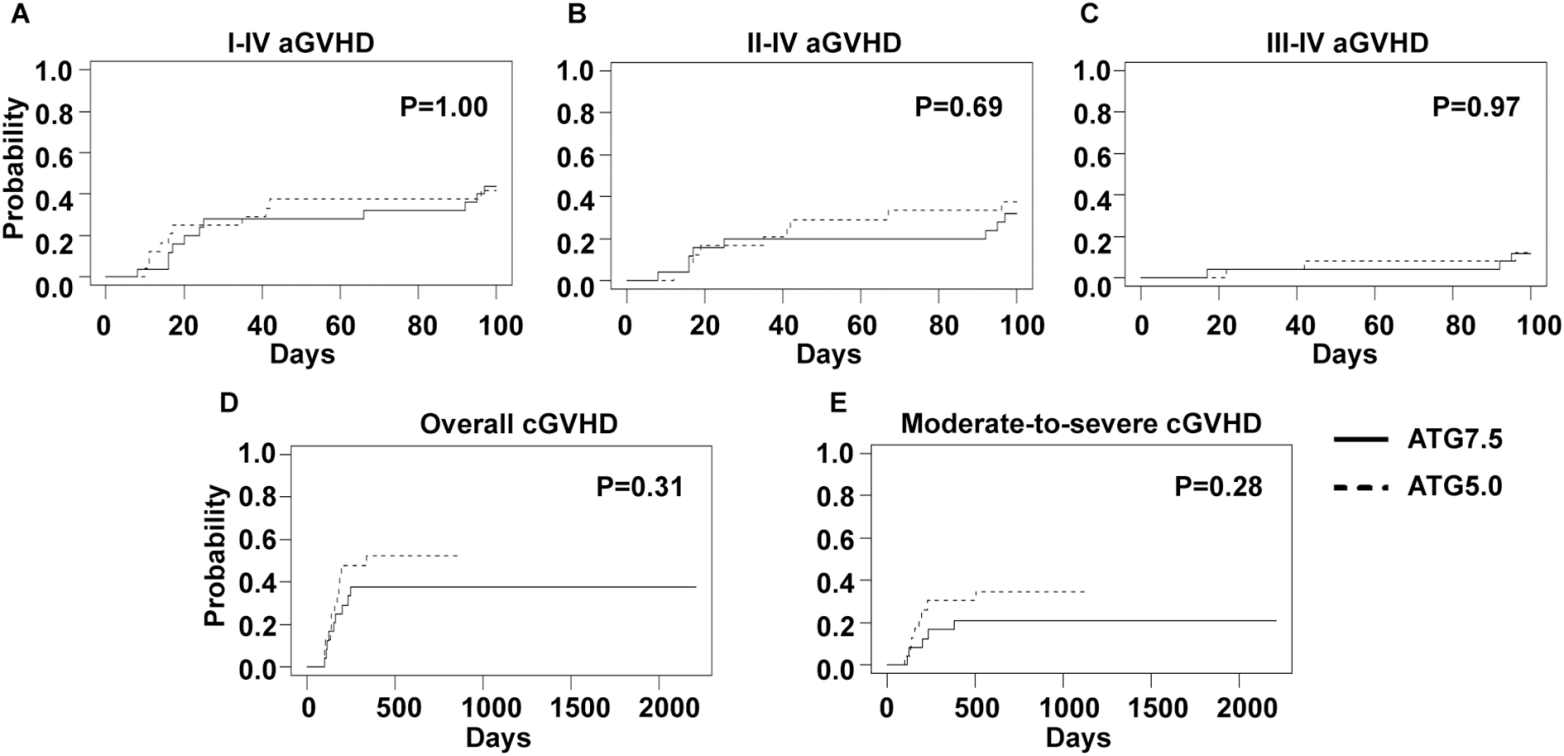
GVHD incidences of 7.5 mg/Kg and 5 mg/Kg ATG groups. Cumulative incidences of I-IV **(A)**, II-IV **(B)** and III-IV **(C)** acute GVHD and overall **(D)** and moderate-to-severe **(E)** chronic GVHD are shown.

In this study, two total ATG doses (7.5 and 5 mg/Kg) were applied in the conditioning regimen. However, no significant difference was observed regarding the CIs of grade I-IV (44.0% versus 41.7%, p=1.00), II-IV (32.0% versus 37.5%, p=0.69) or III-IV (12.0% versus 12.5%, p=0.97) acute GVHD (**Figures 3A–C**). The CIs of overall (37.5% versus 52.2%, p=0.31) and moderate-to-severe (20.8% versus 34.8%, p=0.28) chronic GVHD were similar as well (**Figures 3D, E**).

**Figure 4:**
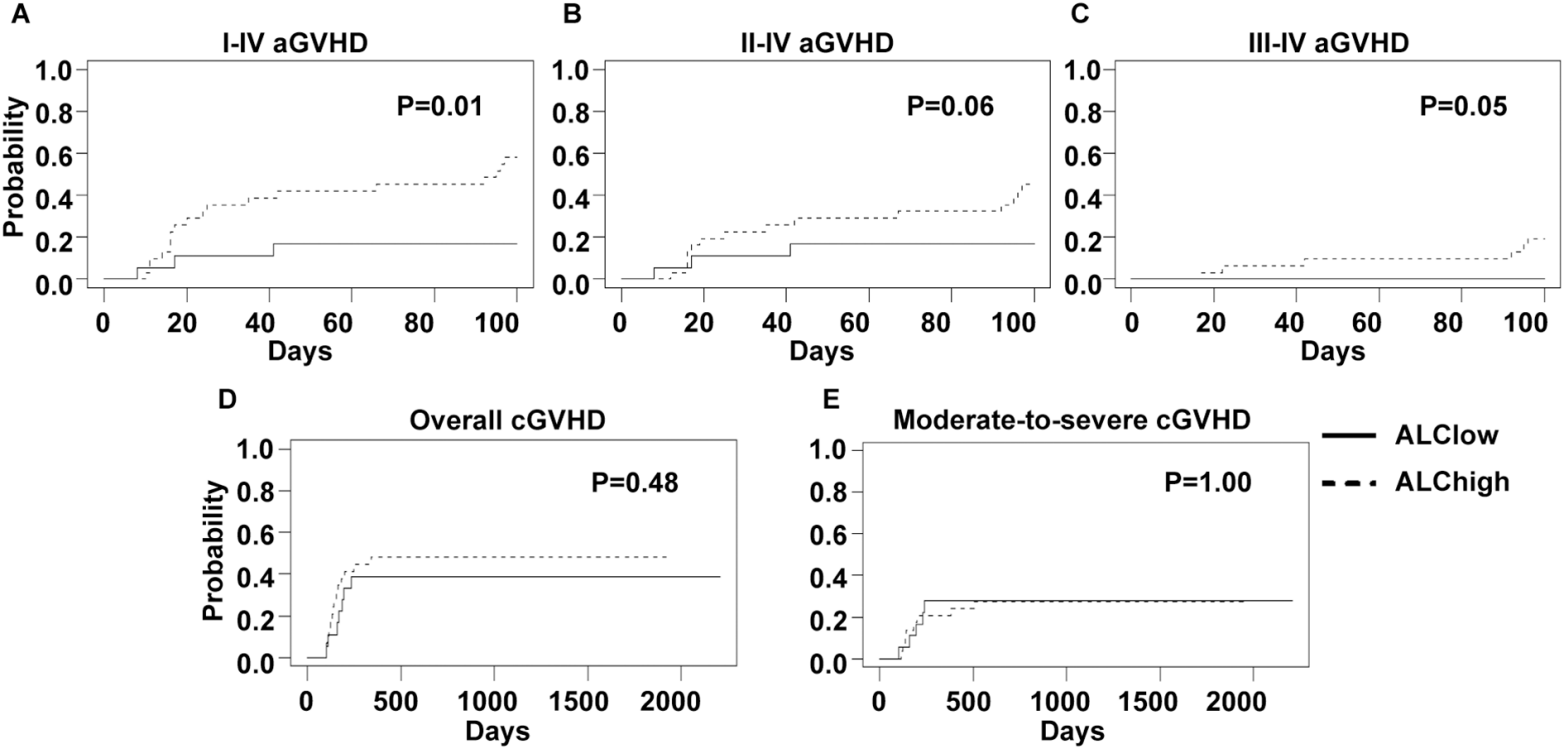
GVHD incidences of low and high ALC groups. An optimal ALC cutoff point of 0.585×10^9^/L, calculated by ROC analysis, was used to divide all patients into low and high ALC groups. Cumulative incidences of I-IV **(A)**, II-IV **(B)** and III-IV **(C)** acute GVHD and overall **(D)** and moderate-to-severe **(E)** chronic GVHD of two ALC groups are shown.

As absolute lymphocyte count on the first day of ATG administration (pre-ATG ALC) was reported as a risk factor for GVHD in the literature, we also explored its effect on GVHD incidences. The median value of pre-ATG ALC was 0.83×10^9^/L (range, 0.01-2.48×10^9^/L) and showed no significant difference between 7.5 mg/Kg and 5.0 mg/Kg ATG groups (median, 0.90×10^9^/L versus 0.76×10^9^/L, *p*=0.73). Then, an optimal pre-ATG ALC cutoff value of 0.585×10^9^/L for grade I-IV and II-IV acute GVHD was calculated using ROC analysis. With this cutoff value, the whole cohort was divided into low (18 patients) and high (31 patients) pre-ATG ALC groups. Low ALC group showed lower CIs of grade I-IV (16.7% versus 58.0%, *p*=0.01), II-IV (16.7% versus 45.1%, *p*=0.06) and III-IV (0 versus 19.4%, *p*=0.05) acute GVHD (**Figures 4A–C**). However, CIs of chronic GVHD showed no significant difference between two ALC groups (**Figures 4D, E**).

Furthermore, we analyzed the impact of different ATG doses and pre-ATG ALC levels on OS, DFS, NRM, CIR and CMV/EBV reactivation (**Supplementary Figures 1, 2**), and no significant differences were observed. The impact of disease status at transplantation (CR1 and non-CR1) and stem cell source (bone marrow included or not) on GVHD and survival was also analyzed. The patients in CR1 showed significant superior 2-year OS (77.1% versus 43.8%, *p*=0.03) and DFS (65.6% versus 31.3%, *p*=0.004) with similar GVHD incidences compared to those in non-CR1 (**Supplementary Figure 3**). The inclusion of bone marrow as stem cell source did not result in significant difference in GVHD incidence and survival (**Supplementary Figure 4**).

## 4 Discussion

ATG and PTCy are two main GVHD-prophylaxis strategies for unmanipulated haplo-HSCT. controversies still exist whether ATG or PTCy brings better survival after transplant (15–17). Alternatively, many transplant centers attempted to combine ATG and PTCy to further optimize their GVHD-prophylaxis regimens, in which various doses and timings of ATG and PTCy were applied (6, 13, 18). In the present study, we report the low-dose ATG/PTCy regimen for haplo-HSCT in our center and the data are comparable to those in the literature, in which ATG and/or PTCy were used for GVHD prophylaxis, regarding GVHD and survival (4, 17–20).

In late 2020, we modified the ATG dose of our protocol from 7.5 mg/Kg to 5.0 mg/Kg, based on the results of a multicenter randomized study reported by Lin et al. In this study, two doses (7.5 mg/kg and 10 mg/kg) of ATG without PTCy were compared in haplo-HSCT and 7.5 mg/kg ATG resulted in reduced EBV/CMV infections without increased incidence of GVHD (20). In our study, the influence of two ATG doses were also analyzed, and no differences were observed regarding incidences of GVHD, CMV/EBV reactivation and survival.

Interestingly, when the whole cohort was divided into two groups by an optimal pre-ATG ALC cutoff point of 0.585×10^9^/L, low ALC group showed lower incidences of acute GVHD. In several studies on population pharmacokinetics of ATG, pre-ATG ALC was determined as an important factor impacting ATG levels (21, 22). As lymphocytes are the main “targets” of ATG, it is reasonable that ALC may influence the ATG level through target-mediated drug disposition (23). In addition, the correlation of pre-ATG ALC with transplant outcomes was also reported by several studies (24–26).

However, conflicting data also exist. Heelan et al. reported a retrospective study which included 111 patients receiving matched unrelated donor HSCT with ATG, and pre-ATG ALC did not correlate with GVHD, relapse or mortality (27). It is noteworthy that, in this study, the range of ALC was 0 - 0.19×10^9^/L, much lower than that in other studies mentioned above. Takahashi et al. reported a novel population pharmacokinetics model for ATG in the HSCT setting with minimal pre-ATG ALC (range 0–0.058×10^9^/L) (23). In this model, influential covariates include ideal body weight, baseline serum albumin level, baseline serum IgG level and CD4^+^ T cell graft dose, rather than ALC. Taken together, it seems that severe lymphopenia may diminish the impact of ALC on ATG level and HSCT outcomes.

In our study, the data favor low pre-ATG ALC level, which correlated with lower acute GVHD and similar survival outcomes, in line with the study of Jamani et al. (22) However, Woo et al. reported that, in the setting of matched related donor HSCT with 4.5 mg/kg total ATG dose, low ALC group (cutoff point 0.50×10^9^/L) had significantly higher NRM and inferior OS, although GVHD incidences were lower (26). We consider that total ATG dose in our regimen is relatively lower than the “optimal” ATG dose, therefore patients may benefit from lower ALC. Therefore, the impact of ALC is supposed to be carefully evaluated for each transplant population and transplant regimen.

In addition, based on our data, it seems that 2.5 mg/kg more ATG dose (7.5 mg/Kg as compared to 5 mg/Kg) is not enough to overcome the increased risk of acute GVHD which high ALC brings. Other strategies should be tried to further optimize GVHD prophylaxis, for example, addition of other cytotoxic drugs, e.g. fludarabine, before ATG infusion to adjust pre-ATG ALC levels, or dose adjustment of PTCy based on pre-ATG ALC levels.

Besides the ATG dose and pre-ATG ALC, we also analyzed the impact of disease status at transplantation and stem cell source on GVHD and survival. Patients in CR1 showed superior survival outcomes compared to those in non-CR1, in line with the literature (19, 28). Bone marrow as stem cell source is generally thought to result in reduced GVHD incidence, though conflicting data still exist (28–30). In our study, inclusion of bone marrow as stem cell source did not show any advantage in GVDH incidence and survival. This might be because bone marrow was applied together with peripheral blood stem cell in most cases, and this is kind of a standard procedure in haplo-HSCT “Beijing protocol”.

Our study has limitations. First, it is a retrospective analysis with a limited number of HSCT patients. Multivariate analysis was not performed and further large prospective clinical trials are needed to confirm the impact of ATG doses and ALC in our low-dose ATG/PTCy regimen. Second, we did not monitor post-transplant immune reconstitution, e.g. CD4^+^/CD8^+^ T cell and NK cell recovery. These objective indicators might better show the impact of ATG doses and ALC. Third, it would be better to monitor ATG levels in this study. Data on ATG pharmacokinetics might provide more evidence to support our findings.

In summary, our study shows that our low-dose ATG/PTCy regimen for GVHD prophylaxis of haplo-HSCT is feasible and pre-ATG ALC has a great impact on acute GVHD incidence in our transplant system. Further attempts to improve GVHD prophylaxis based on ALC levels could be made in the future.

## Data Availability

The original contributions presented in the study are included in the article/**Supplementary Material**. Further inquiries can be directed to the corresponding author.
