# Supplementary material for: Low-dose ATG/PTCy for graft-versus-host disease prevention in haploidentical transplantation: a single-center experience
Source: Front Oncol. 2025 Jun 9;15:1569149. doi: 10.3389/fonc.2025.1569149 (PMC12183090; doi:10.3389/fonc.2025.1569149)
Supplement: Supplementary file 1 [file DataSheet1.pdf]

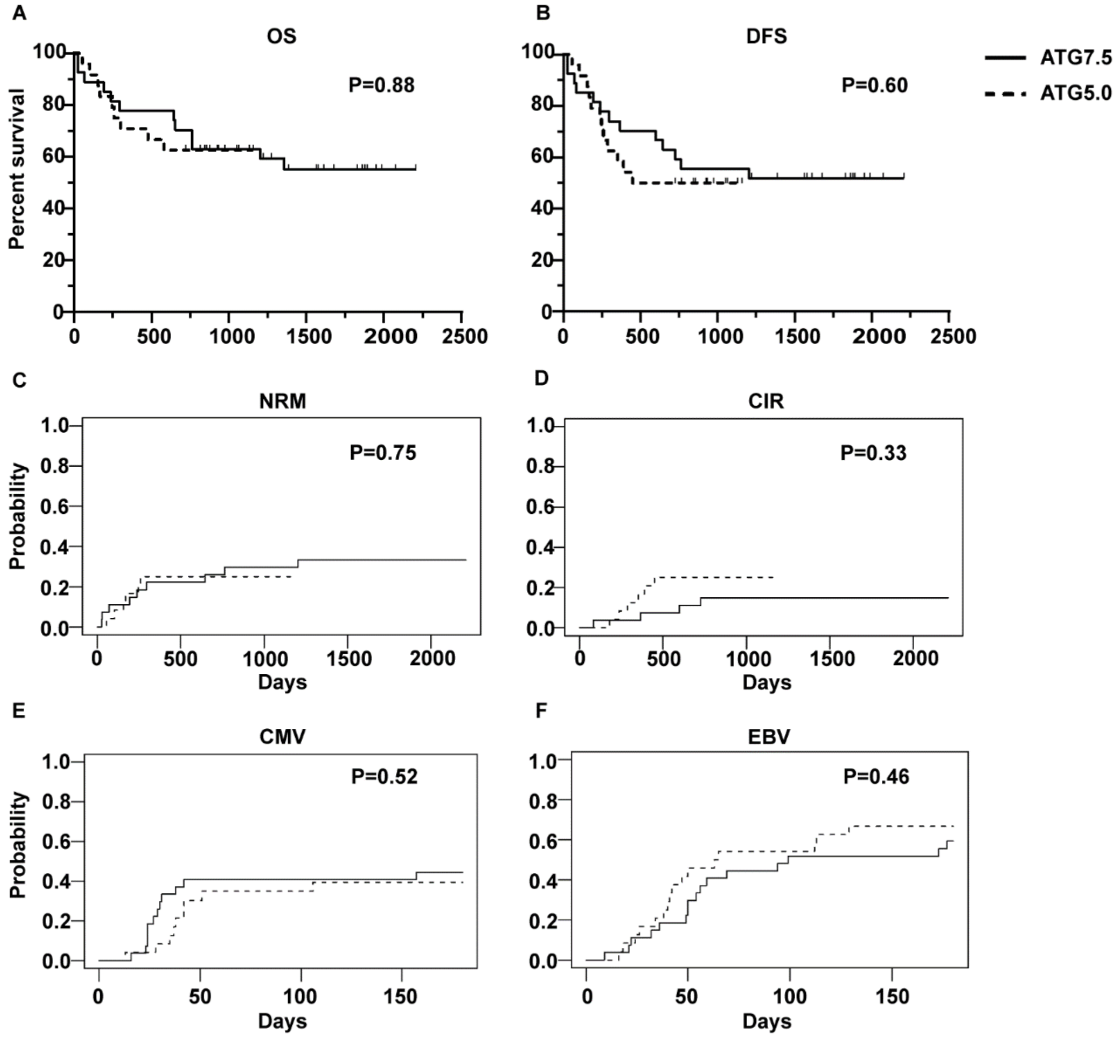

Supplementary Figure 1. Overall survival (OS), disease-free survival (DFS), non-relapse mortality (NRM), cumulative incidence of relapse (CIR) and CMV/EBV reactivations of 7.5 mg/Kg and 5 mg/Kg ATG groups.

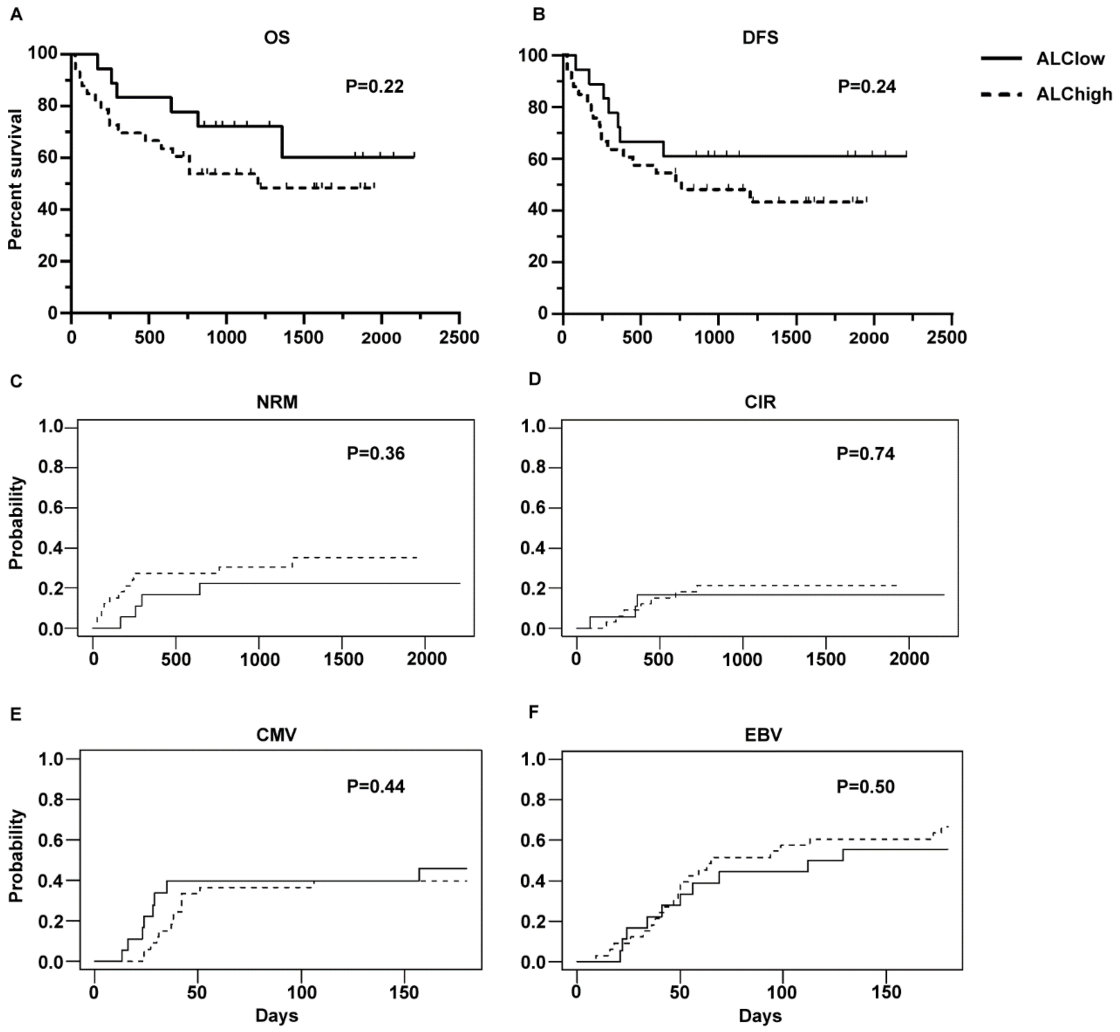

Supplementary Figure 2. Overall survival (OS), disease-free survival (DFS), non-relapse mortality (NRM), cumulative incidence of relapse (CIR) and CMV/EBV reactivations of low and high pre-ATG absolute lymphocyte count (ALC) groups.

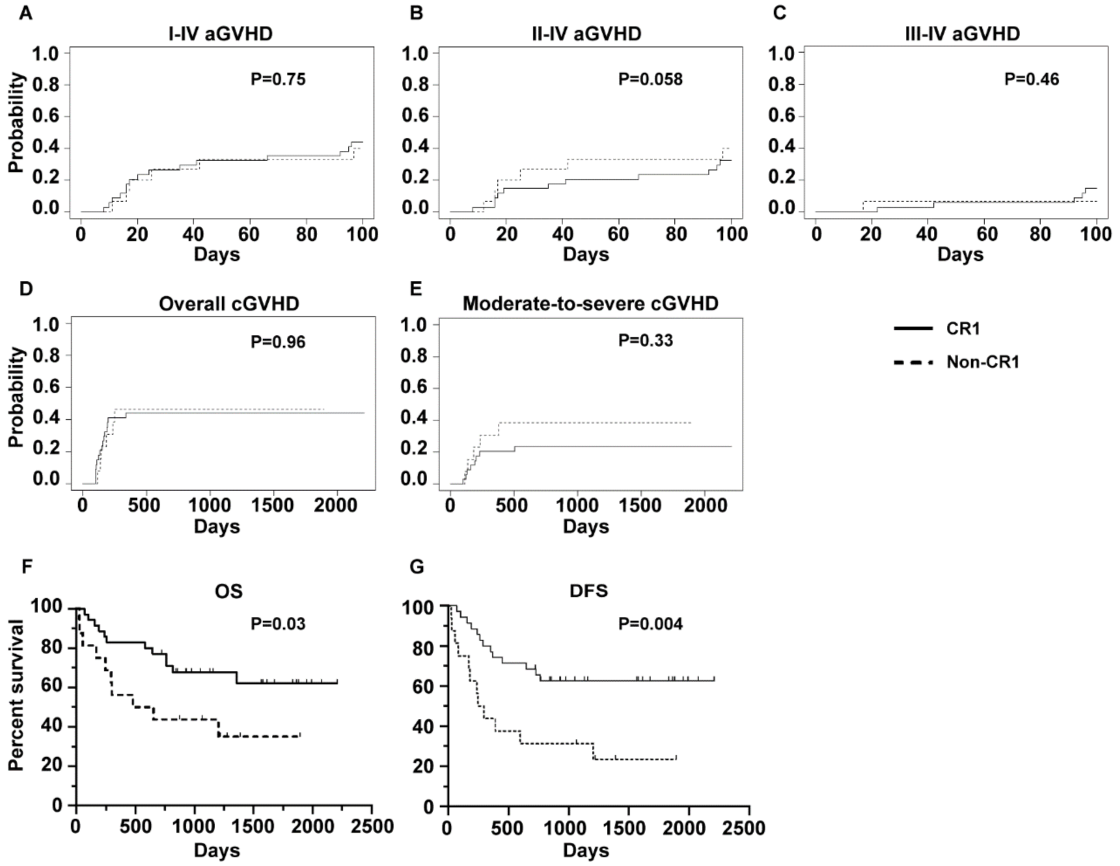

Supplementary Figure 3. GVHD incidences and survival of patients in CR1 and non-CR1. Cumulative incidences of I-IV (A), II-IV (B) and III-IV acute GVHD (C), overall (D) and moderate-to-severe (E) chronic GVHD, overall survival (F) and Disease-free survival (G) of patients in CR1 (n=35) and non-CR1 (n=16) are shown.

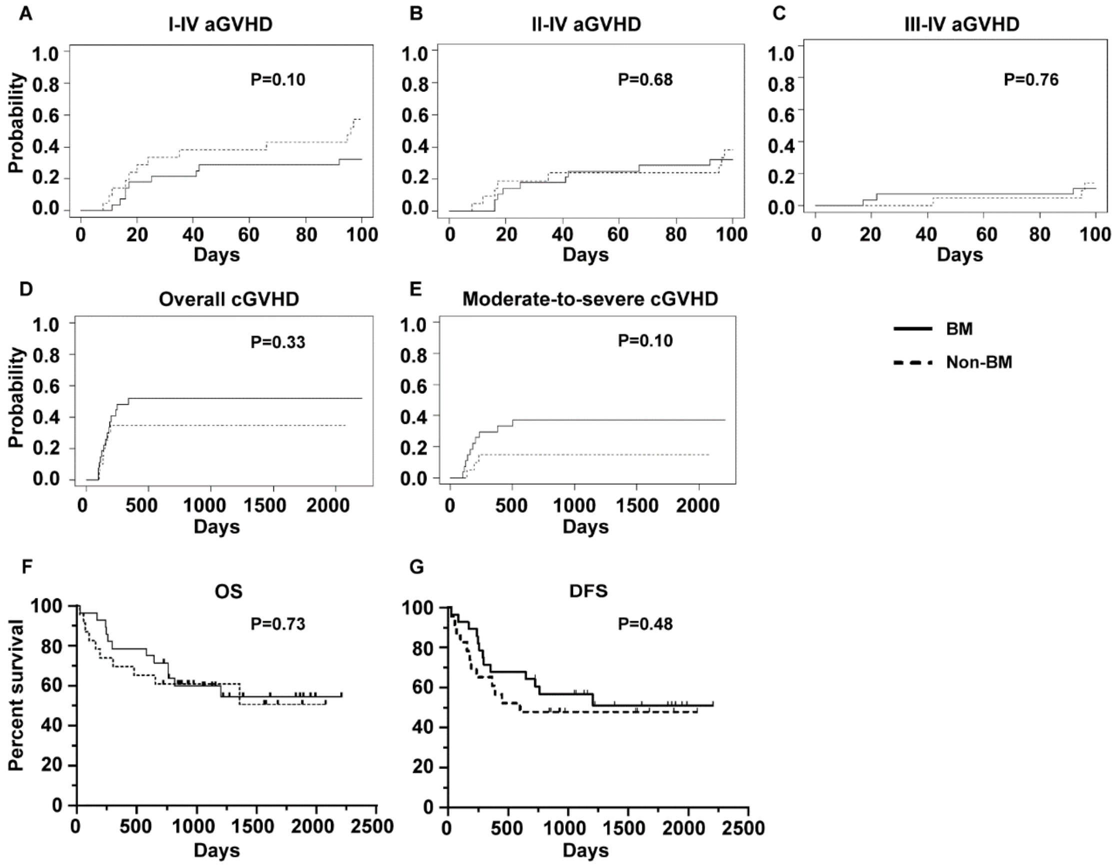

Supplementary Figure 4. GVHD incidences and survival stratified by stem cell source. Patients were categorized into "bone marrow" and "non-bone marrow" groups based on whether bone marrow was included as stem cell source. Cumulative incidences of I-IV (A), II-IV (B) and III-IV acute GVHD (C), overall (D) and moderate-to-severe (E) chronic GVHD, overall survival (F) and Disease-free survival (G) of two groups are shown.
